# 3D Echocardiographic Assessment of Right Ventricular Involvement of Left Ventricular Hypertrabecularization from a New Perspective

**DOI:** 10.3390/jimaging11060181

**Published:** 2025-06-03

**Authors:** Márton Horváth, Kristóf Farkas-Sütő, Flóra Klára Gyulánczi, Alexandra Fábián, Bálint Lakatos, Anna Réka Kiss, Kinga Grebur, Zsófia Gregor, Balázs Mester, Attila Kovács, Béla Merkely, Andrea Szűcs

**Affiliations:** Heart and Vascular Center, Semmelweis University, H-1122 Budapest, Hungary

**Keywords:** hypertrabeculation, right ventricle, 3D echocardiography

## Abstract

Right ventricular (RV) involvement in left ventricular hypertrabeculation (LVNC) remains under investigation. Due to its complex anatomy, assessing RV function is challenging, but 3D transthoracic echocardiography (3D_TTE) offers valuable insights. We aimed to evaluate volumetric, functional, and strain parameters of both ventricles in LVNC patients with preserved left ventricular ejection fraction (EF) and compare findings to a control group. This study included 37 LVNC patients and 37 age- and sex-matched controls. 3D_TTE recordings were analyzed using TomTec Image Arena (v. 4.7) and reVISION software to assess volumes, EF, and global/segmental strains. RV EF was further divided into longitudinal (LEF), radial (REF), and antero-posterior (AEF) components. LV volumes were significantly higher in the LVNC group, while RV volumes were comparable. EF and strain values were lower in both ventricles in LVNC patients. RV movement analysis showed significantly reduced LEF and REF, whereas AEF remained normal. These findings suggest subclinical RV dysfunction in LVNC, emphasizing the need for follow-up, even with preserved EF.

## 1. Introduction

Hypertrabeculation, characterized by excessive trabecular meshwork within the left ventricular (LV) cavity, may be present in up to 20% of the general population, and current recommendations regard asymptomatic individuals without a history of cardiac disease as a physiological variant [1,2].

This morphology may arise in response to chronic volume overload, such as pregnancy, high-level sports activity, or hyperthyroidism, but a distinct subgroup also fulfills the diagnostic criteria of primary left ventricular noncompaction (LVNC) with preserved LV ejection fraction (LV_EF) [3,4,5].

These patients with excessive trabeculation often show increased volumetric and decreased functional parameters, and in some cases, disease progression may lead to impaired LV function, heart failure, arrhythmias, or thromboembolic events [6,7,8].

However, less is known about the role of right ventricular (RV) involvement in this population. While isolated RV noncompaction has been previously described in case reports, recent studies using echocardiography (TTE) and cardiac magnetic resonance imaging (CMR) have investigated the potential biventricular nature of LVNC. These studies revealed RV characteristics resembling those of the LV, namely, increased trabecular mass values, larger volumes, and reduced functional parameters [6,8]. This aspect is particularly studied in subjects with preserved LV_EF, as it allows the assessment of RV involvement without the confounding influence of impaired LV function. Despite these findings, evidence remains heterogeneous and often limited to conventional parameters, underscoring the need for more advanced imaging techniques to better characterize RV mechanics in LVNC.

**Figure 1:**
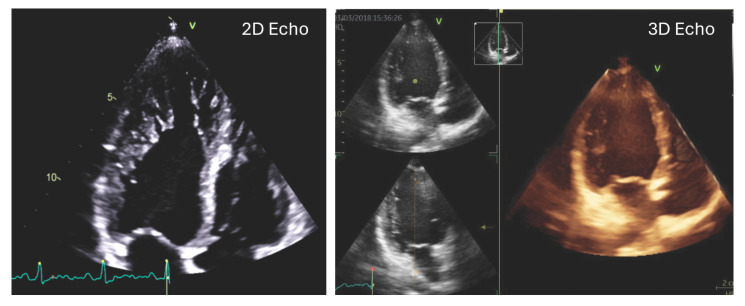
Representative 2D and 3D transthoracic echocardiographic images of a patient with left ventricular hypertrabeculation.

Three-dimensional transthoracic echocardiography (3D TTE) has recently gained attention as a valuable imaging modality that enables comprehensive cardiac assessment by providing spatially detailed views of cardiac structures and motion [9,10]. Although it is less optimal for depicting fine trabecular details, 3D TTE offers high accuracy in quantifying functional and volumetric parameters (Figure 1), as also emphasized in recent guideline recommendations. A novel analytical approach based on 3D TTE facilitates the evaluation of right ventricular (RV) volumes, global function, and directional motion along three orthogonal planes that correspond to the anatomical fiber architecture of the RV [11]. Prior studies across various cardiac conditions have indicated that alterations in RV motion patterns may hold prognostic relevance. Nevertheless, the specific features of RV mechanics, including directional contraction behavior, have not yet been systematically characterized in individuals with LVNC morphology.

Accordingly, our objective was to assess the volumetric, functional, and strain characteristics of both the left and right ventricles, as well as the detailed three-dimensional motion pattern of the RV in individuals with primary LVNC with preserved left ventricular ejection fraction (LV_EF > 50%) using 3D TTE based methods. The findings were subsequently compared to those obtained from an age- and sex-matched control (C) group.

## 2. Materials and Methods

From our registry of LVNC population, we enrolled 37 individuals (males *n* = 22, average age: 40.2 ± 15 years) with preserved left ventricular function who had undergone 3D TTE examinations. To create a control group (C), we also included 37 age- and sex-matched healthy volunteers without known cardiac or systemic disorders (males *n* = 22, average age: 40.3 ± 15 years).

Inclusion criteria for patients encompassed a diagnosis of LVNC confirmed by CMR, meeting both Petersen (ratio of noncompacted to compacted myocardial layer exceeding 2.3 at end-diastole) and Jacquier criteria (trabecular mass greater than 20% of total myocardial mass at end-diastole).

We excluded participants with reduced LV_EF (<50%), coronary artery disease, congenital heart disease, other forms of cardiomyopathies, or significant comorbidities (such as diabetes and untreated hypertension). Additionally, individuals engaged in physical training exceeding 6 h per week and those with images that could not be reliably processed for technical reasons were also excluded. The baseline characteristics of the population are shown in Table 1.

This study involved human participants, all of whom provided written informed consent prior to enrollment. The study protocol adhered to the principles outlined in the Declaration of Helsinki and its subsequent amendments. Ethical approval was granted by the Central Ethics Committee of Hungary.

Three- and two-dimensional transthoracic echocardiographic (3D TTE) studies were performed using a GE Vivid E95 ultrasound system equipped with a 4Vc-D matrix-array transducer (GE Vingmed Ultrasound, Horten, Norway). Full-volume datasets of the left ventricle (LV) and right ventricle (RV) were acquired from an apical four-chamber view using ECG-gated, multibeat acquisition over four cardiac cycles.

All post-processing analyses were conducted by a single experienced operator using commercially available software (4D LV-Analysis (v. 3.1) and 4D RV-Function 2 (v. 2.2.4); TOMTEC Imaging Systems GmbH, Unterschleissheim, Germany). The LV datasets were evaluated first. The software automatically delineated the LV endocardial surface, which was subsequently adjusted manually as needed across long- and short-axis views throughout the cardiac cycle. Speckle tracking was used for the deformation analysis.

For the LV, volumetric indices—including end-diastolic volume (LV_EDV), end-systolic volume (LV_ESV), and stroke volume (LV_SV)—were calculated. Functional metrics such as ejection fraction (LV_EF), global longitudinal strain (LV_GLS), and global circumferential strain (LV_GCS) were also derived. All volumetric data were indexed to body surface area.

Following LV evaluation, the right ventricular (RV) 3D surface model was exported frame by frame throughout the cardiac cycle for further analysis using a dedicated post-processing tool (ReVISION—Right Ventricular Separate Wall Motion Quantification). This custom-designed software performs a vertex-based motion decomposition of the RV mesh model. Among the 2D parameters, the TAPSE and PAP were also calculated.

**Figure 2:**
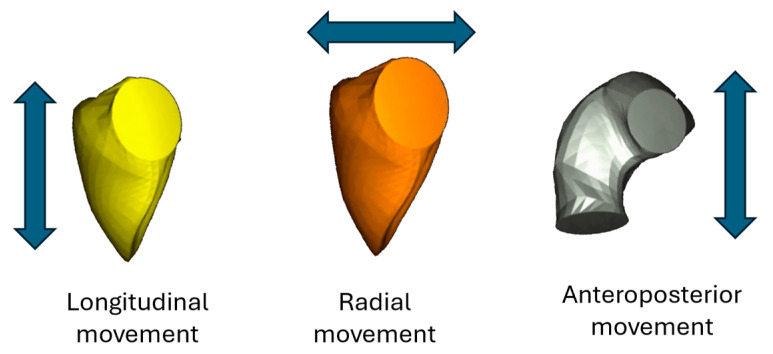
Three-dimensional decomposition of right ventricular contraction into global, longitudinal, radial, and anteroposterior components, visualized using the ReVISION software. This model serves as the basis for the RV motion analysis applied in this study. The arrows indicate the principal direction of contraction for each motion component.

At each time point, directional volume changes were computed using the signed tetrahedron method, allowing motion to be separated along three anatomically defined orthogonal axes. This approach enabled the quantification of the individual contributions from longitudinal, radial, and anteroposterior wall displacement to overall RV volume change (Figure 2).

Standard RV volumetric indices—including end-diastolic volume (RV_EDV), end-systolic volume (RV_ESV), and stroke volume (RV_SV)—were determined, along with global functional parameters such as ejection fraction (RV_EF), global longitudinal strain (RV_GLS), global circumferential strain (RV_GCS), and global area strain (RV_GAS). In addition, the longitudinal (LEF), radial (REF), and anteroposterior (AEF) components of ejection fraction were calculated separately. These were also expressed as ratios relative to RV_EF (LEF/RV_EF, REF/RV_EF, AEF/RV_EF), representing the directional contribution of each motion component to the overall RV function. Normative reference values for these parameters were adopted from Cotella et al. [11].

The normality of data distribution was assessed using the Shapiro–Wilk test. For continuous variables with normal distribution, group comparisons were performed using the independent samples *t*-test; for non-normally distributed data, the Mann–Whitney U test was applied.

Relationships between continuous variables were explored using Pearson’s correlation analysis, with correlation strength interpreted as weak (<0.3), moderate (0.3–0.6), or strong (>0.6).

Interobserver agreement for key 3D echocardiographic parameters (e.g., RV EF, LEF, REF, AEF) was assessed using the two-way random-effects intraclass correlation coefficient (ICC), average measures, with corresponding 95% confidence intervals.

All tests were two-tailed, and a *p*-value < 0.05 was considered statistically significant. Statistical analyses were performed using IBM SPSS Statistics version 28.0.1.0 (IBM Corp., Armonk, NY, USA) and Microsoft Excel for Microsoft 365 (Microsoft Corp., Redmond, WA, USA).

## 3. Results

The interobserver agreement of the LV and RV, as assessed by the ICC, was analyzed in ten randomly selected patients and ten healthy subjects. The results of the interobserver variability test are in Table 2.

First, we analyzed the LV and RV volumetric and functional parameters. The LV volumes were significantly elevated, and the LV_EF and strain parameters were significantly decreased compared to the control group. It is worth noting that, except for the LV strain parameters, which were below the normal cutoff value, all the LV parameters remained within the normal range (Table 2).

Examining the RV, no significant differences were found in volumetric parameters between the two groups, except the RV_EF, RV_GLS, and RV_GAS values of the LVNC group, which were significantly reduced compared to the controls (Table 3).

**Figure 3:**
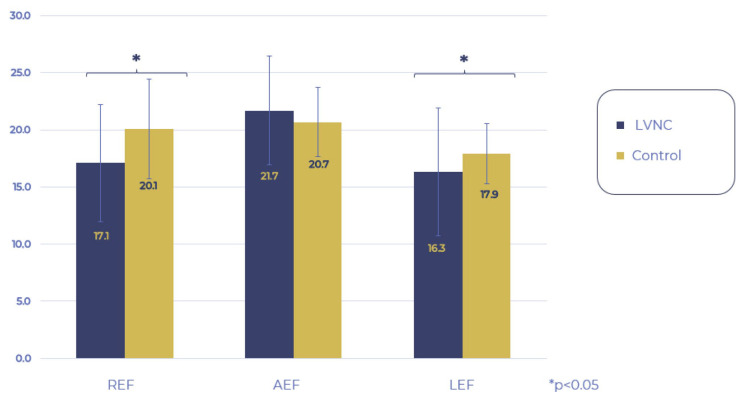
Comparison of 3-way motion components of RV EF. RV: right ventricle, EF: ejection fraction, REF: radial ejection fraction, AEF: anteroposterior ejection fraction, LEF: longitudinal ejection fraction.

**Figure 4:**
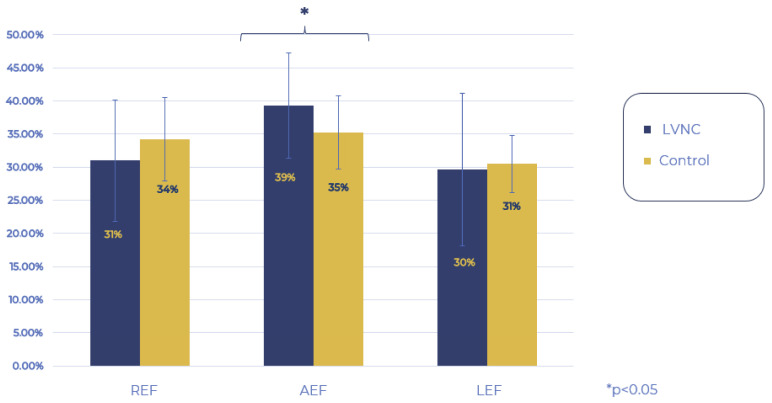
Share of the 3 contraction directions in the global RV RF. REF: radial ejection fraction, AEF: anteroposterior ejection fraction, LEF: longitudinal ejection fraction.

Then, we examined the movement pattern of the RV. When comparing the LEF, REF, and AEF of the LVNC and control groups, the REF and LEF components remained within the normal ranges but were significantly decreased compared to the control group, while AEF contraction remained unchanged. Analyzing the proportion of the three directions regarding the global RV_EF, the AEF had a significantly higher share in the LVNC group compared to the control group, whereas the contribution of REF and LEF contractions did not differ between the two groups (Figure 3 and Figure 4).

Finally, when studying the correlation of volumetric and functional parameters between the LV and RV, we found a moderately strong correlation in the case of EDV and SV, and it is noteworthy that almost all functional parameters showed a strong positive correlation between the two ventricles (Table 4). Then, we examined the relationship of the RV’s three-directional motion components with left and right ventricular volumetric and functional parameters. While among the LV parameters, only the EF correlated with AP contraction, the RV parameters mainly correlated with the directions of contraction, most notably also with the AEF (Table 5 and Table 6).

As a supplementary observation, both the TAPSE and PAP values of LVNC patients were within the normal range (TAPSE: 26 ± 8 mm, PAP: 23 ± 8 Hgmm).

## 4. Discussion

This study used three-dimensional echocardiography to assess the characteristics of the left- and right ventricle, especially the 3D movement pattern of the RV in LVNC subjects with preserved LV_EF compared to healthy controls.

Regarding the LV, our results are in line with previous studies using CMR and cardiac ultrasound; namely, the volumes were increased, and the functional parameters were decreased compared to the controls [6,8]. Studying RV involvement in the LVNC population, our findings align with our previous investigation using 3D_TTE, as we also identified moderately decreased RV function [6]. Previous CMR studies have also revealed subclinical changes, such as elevated RV volumetric parameters and decreased EF and strain values in LVNC individuals with both preserved and decreased LV_EF [8]. This underscores the diagnostic complexity of LVNC, particularly in distinguishing pathologic forms from physiological remodeling [12]. Interestingly, Stämpfli et al. concluded that quantifying RV trabeculation does not facilitate the differentiation between LVNC and healthy subjects, emphasizing the importance of focusing on the ventricle’s functional characteristics [13].

The prognostic value of decreased RV function has been described in several studies in different pathologies [14,15,16]. A study by Wang et al. involving 117 LVNC participants discovered that RV dysfunction was a strong predictor of all-cause mortality, independent of LV function. Moreover, impaired RV_GLS was another independent predictor of mortality as the authors concluded that RV dysfunction is a common and prognostic factor in patients with LVNC and suggested a regular and quantitative assessment of RV function in this population.

A new innovative technique with growing literature has the advantage of specifically characterizing RV function as it provides a complex 3D movement pattern of the RV. In this study, we found AEF-dominated compensation in the LVNC group compared to the controls.

In contrast to our investigation, Surkova and Kovács et al. found that in the case of decreasing LV_EF caused by various etiology, the LEF and AEF components of the global RV contraction showed notable decreases with a concurrent increase in the REF component to maintain the RV_EF. This mechanism could be in the background, as suggested by previous CMR studies reporting slightly decreased but still normal RV_EF in LVNC patients with reduced LV_EF.

In a study with a healthy population, Lakatos et al. found the proportion of the three contraction directions balanced [17]. This publication also revealed that in patients with reduced LV function caused by various etiology, the REF compensation dominated with the early reduction in LEF and AEF, and these findings were also confirmed by other large case–control studies [18,19]. On the contrary, in pathologies associated with elevated RV strain, e.g., pulmonary embolism and atrial septal defect, longitudinal compensation was particularly pronounced independently from the LV function [17]. Tokodi et al. also examined patients’ RV movement patterns before and after mitral valve replacement (MVR), and they found LEF dominated before MVR, which was turned into a radial compensation in the immediate postoperative period. Interestingly, after three months, the REF/RV_EF ratio was similar to that of healthy subjects. This study also suggests that preoperative 3D_TTE parameters may be prognostic for postoperative RV dysfunction [6].

Among individuals with normal RV ejection fraction, the AEF emerged as a significant and independent predictor of adverse outcomes [18]. Since AEF was normal in our study population, it may indicate a good prognosis in LVNC subjects with preserved ejection fraction. Moreover, Gregor et al. found the global RV function of LVNC patients to be preserved regardless of the LVEF being reduced or preserved, which might suggest a longer RV compensation in LVNC compared to other heart diseases.

In summary, this AEF-dominated movement has not been documented previously in the LVNC population; moreover, it differs from the RV movement patterns described in other diseases; thus, this might be a disease-specific pattern. Thus, our findings highlight the potential clinical relevance of 3D echocardiographic analysis. The identification of this subclinical RV dysfunction would serve as an early marker of the progression of this condition. Detecting these compensatory patterns may help to identify patients who could benefit from closer follow-up or additional diagnostic imaging, even in the absence of overt clinical deterioration. This directional analysis, extending beyond conventional 2D metrics, could support individualized risk stratification in line with contemporary ESC recommendations. Larger studies are warranted to confirm these observations and clarify the prognostic value of AEF in this population.

### Limitations

The sample size was relatively small, reflecting the rarity of primary LVNC and the strict inclusion criteria. Nevertheless, the subgroup with preserved EF was derived from a well-established single-center registry, and future analyses will include patients with reduced LV function to investigate the impact of impaired EF on RV motion patterns.

The cross-sectional design limits conclusions regarding the temporal evolution or prognostic significance of the observed RV motion characteristics. However, longitudinal follow-up of this cohort is ongoing and will allow for serial 3D echocardiographic assessments.

While 3D echocardiography is increasingly adopted in clinical practice, its accuracy remains dependent on image quality and acoustic windows. In this study, only datasets with sufficient image quality were included to ensure reliable quantification. Additionally, the lack of laboratory data limits our ability to correlate functional findings with systemic biomarkers.

## 5. Conclusions

This study investigated LV and RV characteristics and RV motion patterns of the LVNC population with preserved LV_EF using 3D echocardiography.

The LV volumes were significantly increased, and the functional parameters of both chambers were significantly decreased compared to controls. Further investigation of the RV movement revealed decreased REF and LEF with preserved AEF compared to C, indicating a potential disease-specific movement pattern of the LVNC population. This subclinical RV dysfunction with anteroposterior compensation suggests the need for further research to confirm the clinical implications and prognostic value of RV involvement in subjects with LVNC.

These findings may have clinical implications, as they highlight the potential role of advanced 3D echocardiographic analysis in detecting early and subclinical RV dysfunction in LVNC patients with preserved LVEF. Incorporating such assessment into routine practice may facilitate more personalized monitoring and timely intervention, which is in line with contemporary heart failure guidelines.

## Figures and Tables

**Table 1:** Baseline characteristics.

	LVNC	Control	*p*
Study population (*n*)	37	37	ns
Number of women (*n*)	15	15	ns
Age (years)	40.2 ± 15.2	40.3 ± 15.0	ns
LV EF (%)	52.4 ± 3.4	60.1 ± 4.0	<0.01
BSA (m^2^)	1.9 ± 0.3	1.9 ± 0.2	ns

LV: left ventricle, EF: ejection fraction, BSA: body surface area, LVNC: left ventricular hypertrabeculation.

**Table 2:** Interobserver agreement of left and right ventricular parameters.

	LV	RV
**EDV**	0.98	0.96
**EF**	0.91	0.63
**GLS**	0.92	0.59
**GCS**	0.89	0.56

EDV: end-diastolic volume, EF: ejection fraction, GLS: global longitudinal strain, GCS: global circumferential strain.

**Table 3:** LV and RV volumetric and functional parameters.

LV	LVNC	Control	*p*
EDV (i)	76.3 ± 17.8	58.9 ± 10.2	<0.01
ESV (i)	35.9 ± 8.7	23.6 ± 4.8	<0.01
SV (i)	40.4 ± 9.6	35.3 ± 6.1	0.008
EF (%)	52.4 ± 3.4	60.1 ± 4.1	<0.01
GLS (%)	−19.1 ± 2.9	−20.6 ± 2.0	0.003
GCS (%)	−24.2 ± 2.6	−30.2 ± 2.7	<0.01
**RV**	**LVNC**	**Control**	***p***
EDV (i)	56.5 ± 13.9	58.1 ± 12.5	0.589
ESV (i)	25.5 ± 7.6	24.2 ± 6.5	0.473
SV (i)	31.0 ± 8.0	33.9 ± 6.8	0.062
EF (%)	55.1 ± 5.6	58.7 ± 4.2	0.003
GCS (%)	−22.4 ± 6.6	−23.3 ± 3.7	0.725
GLS (%)	−19.3 ± 3.1	−22.0 ± 3.3	<0.01
GAS (%)	−37.2 ± 4.5	−40.6 ± 4.0	<0.01
REF (%)	17.08 ± 5.12	20.1 ± 4.37	0.006
REF/RVEF	0.422 ± 0.12	0.47 ± 0.01	0.077
AEF (%)	21.69 ± 4.77	20.67 ± 3.04	0.067
AEF/RVEF	0.53 ± 0.11	0.489 ± 0.07	0.001
LEF (%)	16.33 ± 5.56	17.91 ± 2.62	0.038
LEF/RVEF	0.41 ± 0.14	0.42 ± 0.06	0.479

LV: left ventricle, RV: right ventricle, EF: ejection fraction, LVNC: left ventricular hypertrabeculation, EDV: end-diastolic volume, ESV: end-systolic volume, SV: stroke volume, GLS: global longitudinal strain, GCS: global circumferential strain, GAS: global area strain, REF: radial ejection fraction, AEF: anteroposterior ejection fraction, LEF: longitudinal ejection fraction.

**Table 4:** Correlations between left and right ventricular parameters.

		LV EDV (i)	LV ESV (i)	LV SV (i)	LV EF	LV GCS	LV GLS
**RV EDV (i)**	r	0.332 *	0.316	0.330 *	−0.144	−0.021	0.036
**RV ESV (i)**	r	0.214	0.245	0.176	−0.32	0.132	0.183
**RV SV (i)**	r	0.375 *	0.318	0.408 *	0.055	−0.163	−0.113
**RV EF (%)**	r	0.158	0.061	0.237	0.481 **	−0.340 *	−0.356 *
**RV GCS**	r	−0.009	0.016	−0.031	−0.326 *	0.095	0.171
**RV GLS**	r	−0.012	0.136	−0.145	−0.712 **	0.553 **	0.534 **
**RV GAS**	r	−0.004	0.113	−0.11	−0.577 **	0.391 *	0.448 **

LV: left ventricle, RV: right ventricle, EF: ejection fraction, EDV: end-diastolic volume, ESV: end-systolic volume, SV: stroke volume, GLS: global longitudinal strain, GCS: global circumferential strain, GAS: global area strain. *: *p* < 0.05; **: *p* < 0.01.

**Table 5:** Correlation of the three contraction directions with LV parameters.

		LV EDV (i)	LV ESV (i)	LV SV (i)	LV EF (%)	LV GLS	LV GCS
**REF**	r	0.046	0.083	0.011	−0.041	0.059	0.143
**AEF**	r	0.106	0.068	0.136	0.340 *	−0.207	−0.112
**LEF**	r	0.026	−0.073	0.113	0.234	−0.238	−0.380 *

LV: left ventricle, EF: ejection fraction, EDV: end-diastolic volume, ESV: end-systolic volume, SV: stroke volume, GLS: global longitudinal strain, GCS: global circumferential strain, REF: radial ejection fraction, AEF: anteroposterior ejection fraction, LEF: longitudinal ejection fraction. *: *p* < 0.05.

**Table 6:** Correlation of the three contraction directions with RV parameters.

		RV EDV (i)	RV ESV (i)	RV SV (i)	RV EF (%)	RV GLS	RV GCS	RV GAS
**REF**	r	−0.044	−0.196	0.112	0.357 *	−0.498 **	−0.043	−0.236
**AEF**	r	−0.164	−0.412 *	0.106	0.340 *	0.588 **	−0.771 **	−0.28
**LEF**	r	0.023	−0.053	0.09	0.234	0.179	0.571 **	−0.431 **

RV: right ventricle, EF: ejection fraction, EDV: end-diastolic volume, ESV: end-systolic volume, SV: stroke volume, GLS: global longitudinal strain, GCS: global circumferential strain, GAS: global area strain, REF: radial ejection fraction, AEF: anteroposterior ejection fraction, LEF: longitudinal ejection fraction. *: *p* < 0.05; **: *p* < 0.01.

## Data Availability

The data presented in this study are available on reasonable request from the corresponding author. The data are not publicly available due to privacy or ethical restrictions.

## Abbreviations

The following abbreviations are used in this manuscript:

3D TTE	Three-Dimensional Transthoracic Echocardiography
AEF	Anteroposterior Ejection Fraction
BSA	Body Surface Area
CMR	Cardiac Magnetic Resonance Imaging
C	Control
ECG	Electrocardiogram
EDV	End-Diastolic Volume
EF	Ejection Fraction
ESV	End-Systolic Volume
GAS	Global Area Strain
GCS	Global Circumferential Strain
GLS	Global Longitudinal Strain
ICC	Intraclass Correlation Coefficient
LEF	Longitudinal Ejection Fraction
LV	Left Ventricle
LVNC	Left Ventricular Hypertrabeculation
LV_EDV	Left Ventricular End-Diastolic Volume
LV_EF	Left Ventricular Ejection Fraction
LV_ESV	Left Ventricular End-Systolic Volume
LV_GCS	Left Ventricular Global Circumferential Strain
LV_GLS	Left Ventricular Global Longitudinal Strain
LV_SV	Left Ventricular Stroke Volume
MVR	Mitral Valve Replacement
REF	Radial Ejection Fraction
RV	Right Ventricle
RV_EDV	Right Ventricular End-Diastolic Volume
RV_EF	Right Ventricular Ejection Fraction
RV_ESV	Right Ventricular End-Systolic Volume
RV_GAS	Right Ventricular Global Area Strain
RV_GCS	Right Ventricular Global Circumferential Strain
RV_GLS	Right Ventricular Global Longitudinal Strain
RV_SV	Right Ventricular Stroke Volume
SPSS	Statistical Package for the Social Sciences

## References

[1] Kawel N., Nacif M., Arai A.E., Gomes A.S., Hundley W.G., Johnson W.C., Prince M.R., Stacey R.B., Lima J.A., Bluemke D.A. (2012). Trabeculated (noncompacted) and compact myocardium in adults: The multi-ethnic study of atherosclerosis. Circ. Cardiovasc. Imaging.

[2] McDonagh T.A., Metra M., Adamo M., Gardner R.S., Baumbach A., Böhm M., Burri H., Butler J., Čelutkienė J., Chioncel O. (2023). 2023 Focused Update of the 2021 ESC Guidelines for the diagnosis and treatment of acute and chronic heart failure: Developed by the task force for the diagnosis and treatment of acute and chronic heart failure of the European Society of Cardiology (ESC) with the special contribution of the Heart Failure Association (HFA) of the ESC. Eur. Heart J..

[3] D’Ascenzi F., Solari M., Anselmi F., Cavigli L., Spadaccio C., Focardi M., Di Giacinto B., Barbieri E., Sarto P., Biffi A. (2021). A contemporary insight into the right ventricle structure and function in athletes. Eur. J. Prev. Cardiol..

[4] Jacquier A., Thuny F., Jop B., Giorgi R., Cohen F., Gaubert J.Y., Vidal V., Bartoli J.M., Habib G., Moulin G. (2010). Measurement of trabeculated left ventricular mass using cardiac magnetic resonance imaging in the diagnosis of left ventricular non-compaction. Eur. Heart J..

[5] Petersen S.E., Jensen B., Aung N., Friedrich M.G., McMahon C.J., Mohiddin S.A., Pignatelli R.H., Ricci F., Anderson R.H., Bluemke D.A. (2023). Excessive Trabeculation of the Left Ventricle. JACC Cardiovasc. Imaging.

[6] Horváth M., Farkas-Sütő K., Fábián A., Lakatos B., Kiss A.R., Grebur K., Gregor Z., Mester B., Kovács A., Merkely B. (2023). Highlights of right ventricular characteristics of left ventricular noncompaction using 3D echocardiography. IJC Heart Vasc..

[7] Jefferies J.L. (2021). Left Ventricular Noncompaction Cardiomyopathy: New Clues in a Not So New Disease?. J. Am. Heart Assoc..

[8] Kiss A.R., Gregor Z., Popovics A., Grebur K., Szabó L.E., Dohy Z., Kovács A., Lakatos B.K., Merkely B., Vágó H. (2022). Impact of Right Ventricular Trabeculation on Right Ventricular Function in Patients With Left Ventricular Non-compaction Phenotype. Front. Cardiovasc. Med..

[9] Oechslin E., Jenni R. (2018). Left ventricular noncompaction: From physiologic remodeling to noncompaction cardiomyopathy. J. Am. Col. Cardiol..

[10] Surkova E., Kovács A., Tokodi M., Lakatos B.K., Merkely B., Muraru D., Ruocco A., Parati G., Badano L.P. (2021). Contraction Patterns of the Right Ventricle Associated with Different Degrees of Left Ventricular Systolic Dysfunction. Circ. Cardiovasc. Imaging.

[11] Cotella J.I., Kovacs A., Addetia K., Fabian A., Asch F.M., Lang R.M. (2024). Three-dimensional echocardiographic evaluation of longitudinal and non-longitudinal components of right ventricular contraction: Results from the World Alliance of Societies of Echocardiography study. Eur. Heart J. Cardiovasc. Imaging.

[12] Lang R.M., Badano L.P., Mor-Avi V., Afilalo J., Armstrong A., Ernande L., Flachskampf F.A., Foster E., Goldstein S.A., Kuznetsova T. (2015). Recommendations for cardiac chamber quantification by echocardiography in adults: An update from the American Society of Echocardiography and the European Association of Cardiovascular Imaging. J. Am. Soc. Echocardiogr..

[13] Stämpfli S.F., Gotschy A., Kiarostami P., Özkartal T., Gruner C., Niemann M., Manka R., Tanner F.C. (2022). Right ventricular involvement in left ventricular non-compaction cardiomyopathy. Cardiol. J..

[14] Antoni M.L., Scherptong R.W., Atary J.Z., Boersma E., Holman E.R., van der Wall E.E., Schalij M.J., Bax J.J. (2010). Prognostic value of right ventricular function in patients after acute myocardial infarction treated with primary percutaneous coronary intervention. Circ. Cardiovasc. Imaging.

[15] Carluccio E., Biagioli P., Alunni G., Murrone A., Zuchi C., Coiro S., Riccini C., Mengoni A., D’antonio A., Ambrosio G. (2018). Prognostic Value of Right Ventricular Dysfunction in Heart Failure With Reduced Ejection Fraction. Circ. Cardiovasc. Imaging.

[16] Śpiewak M., Małek Ł.A., Petryka J., Mazurkiewicz Ł., Werys K., Biernacka E.K., Kowalski M., Hoffman P., Demkow M., Miśko J. (2012). Repaired tetralogy of fallot: Ratio of right ventricular volume to left ventricular volume as a marker of right ventricular dilatation. Radiology.

[17] Lakatos B.K., Nabeshima Y., Tokodi M., Nagata Y., Tősér Z., Otani K., Kitano T., Fábián A., Ujvári A., Boros A.M. (2020). Importance of Nonlongitudinal Motion Components in Right Ventricular Function: Three-Dimensional Echocardiographic Study in Healthy Volunteers. J. Am. Soc. Echocardiogr..

[18] Shiida K., Ujvári A., Lakatos B.K., Tokodi M., Kosztin A., Veres B., Schwertner W., Kovács A., Fábián A., Merkely B. (2022). Tricuspid regurgitation and right ventricular contraction pattern in heart failure with reduced ejection fraction: A 3D echocardiography study. Cardiol. Hung..

[19] Tokodi M., Németh E., Lakatos B.K., Kispál E., Tősér Z., Staub L., Rácz K., Soltész Á., Szigeti S., Varga T. (2020). Right ventricular mechanical pattern in patients undergoing mitral valve surgery: A predictor of post-operative dysfunction?. ESC Heart Fail..

